# Effectiveness of a Community-Based Intervention to Increase Supermarket Vendors’ Compliance with Age Restrictions for Alcohol Sales in Spain: A Pilot Study

**DOI:** 10.3390/ijerph17165991

**Published:** 2020-08-18

**Authors:** Mariàngels Duch, Elena Gervilla, Montse Juan, Clarisse Guimarães, Maite Kefauver, Tobias H. Elgán, Johanna Gripenberg, Zara Quigg

**Affiliations:** 1 European Institute of Studies on Prevention, Rambla, 15 (2º-3º), 07003 Palma, Spain; mduch@irefrea.org (M.D.); mjuan@irefrea.org (M.J.); mkefauver@irefrea.org (M.K.); 2 Balearic Islands Health Research Institute, Carretera de Valldemossa, 79, 07120 Palma, Spain; 3 Faculty of Psychology, University of the Balearic Islands, Carretera de Valldemossa, km 7.5, 07122 Palma, Spain; clarisseparenteguimaraes@gmail.com; 4 STAD, Centre for Psychiatry Research, Department of Clinical Neuroscience, Karolinska Institutet, & Stockholm Health Care Services, Region Stockholm, Norra Stationsgatan 69, 11364 Stockholm, Sweden; tobias.elgan@ki.se (T.H.E.); johanna.gripenberg@ki.se (J.G.); 5 Public Health Institute, Liverpool John Moores University, Exchange Station, Tithebarn Street, Liverpool L2 2QP, UK; Z.A.Quigg@ljmu.ac.uk

**Keywords:** alcohol, adolescents, vendors’ compliance, community-based intervention, multicomponent intervention, community mobilization, mystery shopping

## Abstract

In Spain the legal age to buy alcohol is 18 years. However, official surveys show that minors perceive alcohol availability to be easy. This paper describes the impacts of a community-based intervention to increase vendors’ compliance with age limits regarding alcohol sales in supermarkets. The aim of this study was to explore the association between implementation of a multicomponent intervention to reduce adolescents’ alcohol use and sale of alcohol to minors in the city of Palma (Spain). Twenty trained adolescents (14–17 years old) conducted 138 alcohol test purchases in nine supermarket chains in August 2018 (baseline; *n* = 73) prior to the intervention, and again in January 2020 (follow-up; *n* = 65). Analysis was conducted according to three levels of intervention implemented across the supermarkets: (i) personnel from the supermarkets’ Human Resources or Corporate Social Responsibility teams received alcohol service training as trainers (i.e., community mobilization); (ii) managers and vendors training by the capacitated trainers; and (iii) no training of managers or vendors (i.e., control group). In the supermarkets that completed the Training of Trainers and the vendors’ training program, average sales decreased significantly from 76.9% in 2018 to 45.5% in 2020, asking for the age of the shopper significantly increased from 3.8% to 45.4%, and asking for proof of age significantly increased from 15.4% to 72.7%. Additionally, a statistically significant increase was observed in the visibility of prohibition to sell alcohol to minors’ signs, from 61.5% to 100%. No statistically significant differences were found for the Training of Trainers intervention alone nor in the control group. In conclusion, community mobilization combined with staff training is associated with significant increases in supermarket vendors’ compliance with alcohol legislation in Spain.

## 1. Introduction

Alcohol is the most prevalent substance used among adolescents and young adults [1]. Some studies have found a relationship between alcohol use and short- and long-term negative outcomes such as school dropout, risky sexual behavior, traffic accidents, assaults, injuries/violence, future substance use/abuse and suicide, and substantial health loss [2,3,4,5,6].

Literature indicates that a community-based approach, i.e., actively involving, engaging, and mobilizing communities, is the most effective approach to prevent youngsters’ drug use [7,8,9]. Hence, it is vital that policy makers reinforce current alcohol control policies and enhance community-based prevention programs [6]. In this sense, the *Stockholm Prevents Alcohol and Drug Problems* (STAD) model is a proven strategy that addresses alcohol sales to minors [10] and youngsters’ binge drinking and overserving [11] in nightlife contexts through both formal and informal control measures and community support for implementation of such measures. A study on the cost-effectiveness of the STAD model showed a cost saving ratio of 1 to 39 [12]. The STAD approach combines community mobilization and collaboration with stakeholders, staff training, policy work, and improved law enforcement (formal and informal). As part of the actions involved in this strategy, controlling alcohol availability is key to sensitize and engage communities and reduce the impact that alcohol availability has on youngsters’ alcohol consumption and the related health impacts [13]. Moreover, studies show that access to alcohol can be reduced by setting age limits for access [14]. Other control measures to restrict availability are not over-serving alcohol beverages, rigorous enforcement of legislation, pricing policies, and restriction on alcohol marketing and advertising strategies, etc. [15].

Although current legislation in Spain sets 18 years as the minimum legal age to access alcohol, the majority of underage youth report easy access to alcohol in official school surveys [9]. The elaboration and passing of a comprehensive alcohol law would significantly improve public health but several attempts have been rejected up to four times. Four executives, from both main parties, have tried and failed to regulate the sector in relation to—but not exclusively—advertising and access of minors to alcohol. The gaps in current legislation facilitate access and difficult supervision and enforcement. Consequently, adolescents can get alcohol through many ways and from many places including convenience stores and supermarkets, bars and clubs, or social sources (i.e., friends or siblings with the legal age to purchase it) [4]. Alcohol availability can be prevented by setting minimum age limits, but the effectiveness of such control measures depends on the degree of compliance [16] and vendors do not always comply with the legislation [17,18]. A significant number of vendors in retail establishments fail to properly verify the clients’ age and sell alcohol to minors, emphasizing the need for interventions that can reduce youth access to alcohol through commercial sources [19,20,21,22,23].

Among the existing strategies for preventing underage sales of alcoholic beverages, law enforcement compliance checks are one effective approach, but they are expensive to implement [4]. In the absence of dedicated funding and public support, compliance checks are seldom sustained or not conducted frequently enough to have more than short-life effects [19,20].

Although community-level restrictions on alcohol availability to youth have been suggested as positive intervention strategies, few studies have investigated the effects of these kind of interventions on reducing alcohol availability to underage people at the local level [24]. The aim of this study was to decrease alcohol sales to minors in supermarkets by implementing a community-based multicomponent intervention tailored from the aforementioned STAD model. To assess alcohol sales to minors, an alcohol test purchase protocol was developed, and a mystery shopping strategy was implemented to collect data pre- and post-intervention.

## 2. Materials and Methods

### 2.1. Participant Stores

We selected 21 stores which are part of the nine major supermarket chains in the Balearic Islands (Spain). Since the aim of the study was to decrease alcohol sales to minors in supermarkets of the municipality of Palma, supermarkets were not randomized. Instead, research was conducted in Palma where all nine major supermarket chains were represented.

### 2.2. Alcohol Test Purchases

Alcohol test purchases (*n* = 138) were made by trained adolescents (14–17 years old) in the municipality of Palma. Visits to stores took place on Friday evenings (18.00 h to 20.00 h) when the adolescents were asked to buy a bottle of distilled alcoholic beverage (rum), a bottle of cola soft drink, and two bags of snacks; and on Saturday afternoons (12.00 h to 14.00 h) where they were asked to purchase a fermented alcoholic beverage (six bottles of beer) and two bags of snacks. For unsuccessful sales, the minors were instructed to have a non-confrontational attitude, leave the goods at the cashier’s desk, and exit the store and return to the researcher to complete the information sheet. Baseline data (*n* = 73) was collected in August 2018 and post-intervention data (*n* = 65) in January 2020.

On arriving at the target store, trained observers were given a reminder on the script to follow and shoppers received a 20 euros bill. After that, shoppers entered the store while the trained observer waited unobtrusively near the entrance. Once inside, they first searched for any underage alcohol sales warning signs to check if they were clearly visible and then selected the products to purchase and attempted to purchase them at the check-out. After exiting the store, adolescents met with the observers and gave them any purchases, receipts and change and, together, filled out an information form which has a set of questions to facilitate the collection of information regarding the purchase attempt (day, time, store, type of alcohol sale), the store (visibility and placement of the underage alcohol warning sign), and the vendors behavior (asking for age and/or ID card (identification or identity card)).

### 2.3. Mystery Shoppers

Mystery shoppers were identified through a snowball procedure via a group of parents actively engaged in a local family prevention program. Presentations of the project were made to their parents and informed consent was requested from the parents and the children for adolescents’ participation. Training of the adolescents was conducted on alcohol test purchase protocol and collation of information for the purposes of the study. Clear instructions were given on not lying about their age, if asked, and to show their ID card, if requested. In addition, adolescents were instructed to wear casual informal clothing and no make-up or disguising complements/features, so that they would not look older than their real age.

Twenty adolescents (14–17 years of age) participated in the study, ten in the baseline data collection and ten in the post-intervention study. They were grouped in pairs according to their age group (14–15 year of age and 16–17 years of age). Adolescents were accompanied by a trained researcher to observe the purchase attempt and be able to intervene in case any problems arose.

### 2.4. Multicomponent Intervention

The multicomponent intervention tested in the current study consisted of community mobilization, training of trainers, and training of staff at supermarkets. Baseline data from the mystery shopping conducted in August 2018 was first presented to the municipal coordinator of the project (October 2018), and then to the political and technical Commission constituted to prevent adolescents and youngster’s alcohol consumption in open-air public places. The Commission agreed to summon the representatives of the supermarket chains and the business organizations that represent them for data presentation and informal enforcement. During the months of March and April 2019, the municipality organized meetings with each business group and their organizations and global and store-specific data were presented. Finally, in May 2019 the municipality signed an agreement with four business organizations representing the main nine supermarket chains in the city. The agreement included: (i) the designation of a representative to attend a Training of Trainers workshop to facilitate training of the supermarkets staff; (ii) compliance with the legislation regarding underage alcohol sales and warning signs; and, (iii) adoption of the municipal sensitization campaign “A Palma, menors 0,0” (In Palma, minors 0,0) in the stores and marketing materials. In addition, to facilitate community sensitization and mobilization, a media campaign was launched to reach both local and national media (TV, newspapers, social media, etc.). Attention from the media was gained through press releases during the implementation process, prepared by the municipality along with the research team; TV interviews to members of the Municipal Commission; and TV interviews to relevant stakeholders from the Spanish National Plan on Drugs (PNSD) and the European Monitoring Centre for Drugs and Drug Addiction (EMCDDA) for endorsement.

In August 2019, the researchers implemented a Training of Trainers workshop for representatives from the supermarket chains, mainly staff from their Human Resources and Corporative Responsibility and Prevention departments. A total of nine participants representing six supermarket chains—of a total of nine chains invited—attended the training. The three supermarket chains that did not participate in these trainings of trainers have been considered as control group. During the month of September 2019, the supermarket chains were instructed to undertake their in-house trainings and inform on development. Out of these six supermarket chains, just two provided consistent information on the trainings of store staff carried out (they have been considered the ‘Training of Trainers and Vendors Training’ group). The other four supermarket chains, although some informed on having added a specific module on alcohol sales in their regular training programs, have been considered as just participating in the Training of Trainers group since no additional information was provided. The two supermarket chains that participated in the Training of Trainers and conducted their in-house trainings adopted different strategies that will need further analysis in future projects. One conducted six in-house trainings reaching a total of 73 persons from their staff. The other one conducted one training reaching 22 store managers, and through them, a total of 672 check-out cashiers were trained.

### 2.5. Ethical Issues

The study protocol was developed based on similar studies conducted elsewhere [16,25] and following the principles of the Declaration of Helsinki, ensuring the safety of the research team and study venues and consistency in the implementation of the test purchases. As the study subjects were venues and not humans, and to maintain the unobtrusive nature of the study (Palma is a rather small city), formal ethical approval for the study was not obtained and venues were not informed about the test purchases prior to implementation. Study methods ensured that the venue names were not made publicly available and only shared with the Coordinator of the Municipal Commission in charge of presenting the data individually to each supermarket chain for the purpose of intervention development (engagement in the subsequent Training of Trainers and staff trainings).

Regarding the adolescents’ participation in the research as mystery shoppers, two ethical considerations were considered relevant. First, we did not want our study to encourage participants to consume alcohol. Therefore, to identify and select minors for the alcohol purchase tests, parents participating in a family prevention program were contacted and project presented. Through them, we contacted and only selected adolescents who, for different personal reasons (e.g., involved in sport practice) or family reasons (e.g., a family member experiencing/experienced alcohol problems) were truly conscious on the negative effects of alcohol consumption and both the adolescents and their parents were asked for informed consent. Furthermore, prior to each shopping session participants were debriefed on the dangers of alcohol and the researcher reiterated that the results and procedures should not to be shared with anyone else including their friends and classmates after each proof of sale. Second, special attention was placed to prevent and avoid difficult situations with vendors or security personnel. At all times, the trained observers were ready to intervene if necessary and a letter informing on the research purpose was provided to all teams to be presented to store staff if needed. None of these situations were encountered during the implementation of the test purchases.

Regarding the supermarkets involved, although the selected supermarkets were not informed and prior consent was not requested, the social relevance of the research topic (prevention of underage drinking), the inadequacy of conventional research methods (industry accounts vs. adolescents’ experiences), the public nature of the activity (open sight interaction between vendors and customers), and the lack of negative consequences for the stores and vendors (anonymity of stores and vendors preserved in the study) supported the suitability of the research approach, as has been stated in previous projects [26].

## 3. Results

**Figure 1:**
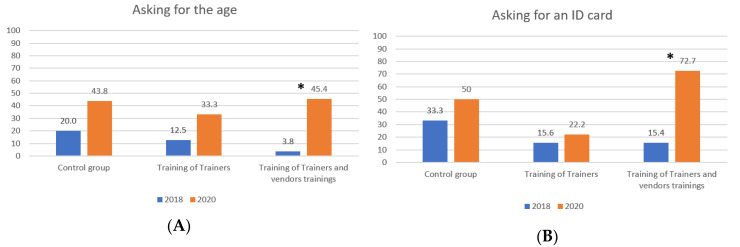
Proportion of supermarket visits by year and type of training. (**A**) Proportion of supermarket visits where minors were asked for their age and (**B**) proportion of supermarket visits where vendors asked for an ID card. * = statistically significant differences were found.

**Figure 2:**
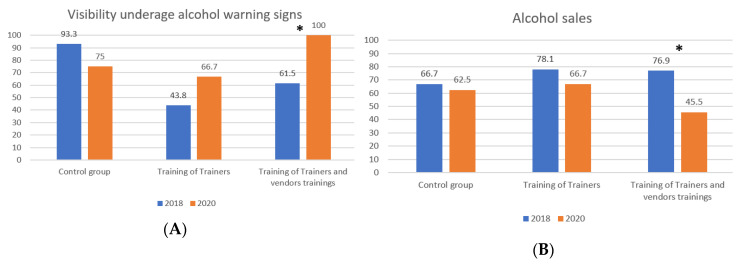
Proportion of supermarket visits by year and type of training. (**A**) Proportion of supermarket visits where underage alcohol warning signs were visible and (**B**) proportion of alcohol sales to minors. * = statistically significant differences were found.

Baseline data was collected in August 2018 (71 purchase attempts) and post-intervention shopping data in January 2020 (67 purchase attempts). Figure 1 shows the proportion of supermarket visits where minors were asked for their age or an ID and Figure 2 shows the proportion of supermarket visits where minors successfully bought alcohol and if underage alcohol warning signs where visible.

Figure 1 and Figure 2 show that between 2018 and 2020 we found a significant change only in the group of supermarkets that conducted the Training of Trainers along with the training of vendors program.

However, we also assessed whether these changes were the same if we had taken into account the age of the minors who were acting as mystery shoppers at the two assessment points. In the baseline data we found differences in alcohol sales by age which showed that the majority of old minors (88.9%, *n* = 40) were able to buy alcohol versus 53.6% of younger minors (*n* = 15). This difference was statistically significant (χ^2^ (1, N = 73) = 11.589; *p* < 0.001). The same happened with asking for an ID card; 39.3% of younger minors were asked to show their ID card (*n* = 11) while only 6.7% (*n* = 3) of older minors were asked to show this identification. This difference was statistically significant (χ^2^ (1, N = 73) = 11.848; *p* < 0.001). We did not find statistically significant differences in asking for the age by group of age in the baseline. In the post-intervention data, we only found statistically differences in asking for an ID card; the majority of younger minors (73.3%, *n* = 11) were asked to show an ID card versus 38% of older minors (*n* = 19). This difference was statistically significant (χ^2^ (1, N = 65) = 5.796; *p* = 0.016). We did not find statistically significant differences in asking for the age and alcohol sales by age (younger and older minors). Since the aforementioned differences were found, we ran the following analyses by group of age.

Table 1 presents the analysis for younger minors (14–15 years old) and shows that we did not find statistically significant differences in any of the four assessed indicators in the results of the proof of sale made in any supermarket, regardless of the intervention group.

Table 2 presents the results of the intervention when older adolescents (16–17 years old) acted as mystery shoppers. Results show that statistically significant differences were found in the intervention group that did the Training of Trainers with vendors trainings. In this group, the frequency of times cashiers asked for the age of the participant adolescents increased from 0% to 43.8% (*p* = 0.007, Fisher’s exact test). Similarly, the proportion of times vendors asked for an ID card significantly increased from 6.3% to 68.8% (χ^2^ (1, N = 32) = 13.333; *p* < 0.001). Finally, the proportion of alcohol sales to minors decreased from 93.8% to 31.3% (χ^2^ (1, N = 32) = 13.333; *p* < 0.001). We did not find statistically significant differences in any of the four assessed indicators in the results of the proof of sale made in the supermarkets who participated in the Training of Trainers nor in those that did not participate in the intervention (control group).

Finally, Table 3 presents the change in the proportion of proof of sales where adolescents saw underage alcohol warning signs. In the Training of Trainers with vendors trainings, we found an increase in the visibility of underage alcohol warning signs from 61.5% to 100% (*p* = 0.001, Fisher’s exact test).

## 4. Discussion

The purpose of this study was to gain a better understanding of potential changes in alcohol availability to minors after a municipal multicomponent intervention consisting of community mobilization and staff training. The results provide supporting evidence that actively engaging industry in prevention efforts results in a reduction in alcohol sales to minors. The media campaign may have had an impact on the outcomes, although no-significant trends have been appraised in the control group or the Training of Trainers group.

Our results are consistent with previous literature which showed that shop owners that received letters summarizing the results of underage mystery shops increased significantly the compliance in their shops, in comparison with shop owners that did not receive the letters [18]. Most of the shop owners interviewed in that study stated that the action most commonly taken after receiving the letter was to bring it to the attention of their staff. Also, in a study of a multicomponent intervention utilizing similar prevention strategies as in the current study, results showed a decrease in alcohol sales to underage people at licensed premises [10].

Mystery shopping can be a part of law enforcement compliance check when the attempt is made by minors under the supervision of law enforcement agents, who can apply a penalty if the alcohol sale is achieved (e.g., UK, Sweden, New Zealand). Mystery shopping interventions can increase alcohol sales compliance and are an effective strategy to include in alcohol availability control prevention programs [4,21]. Studies have shown that requesting ID increases compliance with alcohol legislation [27] and monitoring and feedback on performance is successfully used in a variety of organization settings including alcohol selling establishments in The Netherlands [18,21].

Whereas law enforcement compliance checks have proven to reduce the odds of alcohol sales (US data from the CMDA project (Complying with the Minimum Drinking Age) indicates more than 30% reduction [4]) and they also have a general deterrent effect on nearby establishments, these effects may decay over time unless trainings and formal and informal enforcement measures are in place. Therefore, frequent compliance checks—conducted both by commercial companies and police/law enforcement agencies—are needed to maintain the effects over time with greater probability [4].

Research suggests that there are several factors that may increase the odds of underage alcohol compliance: (1) the use of age verification systems, which allow the cashier to calculate and confirm whether the buyer has the minimum legal age; (2) responsible beverage sales and service programs; and (3) law enforcement checks, especially for those establishments that have been checked themselves or establishments that have one or more neighbors nearby (1–125 m distance) that had an underage alcohol compliance check within the past 90 days [4,10,16,19].

Interestingly, the work carried out with the Training of Trainers has shown several reasons for non-compliance with the legal age limits for alcohol sales. At the company level, issues (such as lack of continuous training and high staff turnover) have been pointed out along with lack of internal/external supervision, lack of resources, and vendors productivity demand. Among staff working in supermarkets, the main reasons include lack of consequences, identification with the customer (age, personal experience, normalization of alcohol use), conflict avoidance (being afraid of intervening), difficulties to estimate the age of the minor when checking the ID, and lack of discipline. Therefore, to enhance compliance it is important to raise awareness on the problem and its consequences and facilitate a multicomponent intervention including community sensitization, training, and formal and informal law enforcement to create a culture of demand and compliance with the regulations. This study, pioneered in Spain, advances evidence on an understudied issue and provides an intervention and evaluation approach that can be easily adopted in other parts of Spain. In Palma, the intervention will be included in the new Municipal Drug Plan to be deployed by the Public Health area in conjunction with the Social Rights and Welfare area and the local Police. Even though prevention education interventions based on personal and social skills and social influence have provided good results among adolescents, their effectivity tends to decrease over time if not sustained by a community-based multicomponent intervention.

Some limitations of this study must be considered. First, the small sample size that reduces the power of the study to detect potential results of the intervention. In this case though, the results on proof of sale are consistent with those presented in national data [28] as well as with the studies carried out in Palma on alcohol consumption in open-air public places that show dangerous alcohol (as well as other drug use) consumption among minors [29,30]. In addition, differences found between pre- and post-intervention data are statistically significant only for the older group of minors (16–17 years); this could be due to the lower representation in the sample for the younger group of minors (14–15 years) or to the fact that they were more easily detected. Second, as explained in Section 2.5 (Ethical Issues), supermarkets were not randomly selected nor allocated to the study groups. Being a study where the main purpose was to create local data to sensitize both municipal agents and supermarket chains, it was considered more important to select four central areas of Palma where representation of all the main supermarket chains was consistent. Moreover, the assessed supermarkets were only a limited sample of places where adolescents can have access to alcohol. Finally, the outcomes may depend on whether the purchase attempts were made by a boy or a girl and/or whether the salesperson was male or female and their own age.

In terms of future research, it would be useful to extend the current findings by examining a larger sample of supermarkets, and to include a randomized sampling method, especially to explore potential differences between the different training strategies used by the supermarket chains who conducted the Training of Trainers plus the training of vendors. Future studies should also collect information on the characteristics of the supermarket (e.g., size), the vendor (e.g., gender, age), and the young person purchasing the alcohol (e.g., gender) to control for addition factors that may be associated with alcohol sales.

## 5. Conclusions

Community mobilization combined with staff training is associated with significant increases in supermarket vendors’ compliance with alcohol legislation in Spain. However, although some supermarket chains show a sales decrease to adolescents along with an increase in proof of age supervision, the results show that compliance level at supermarkets in Palma is still very permissive regarding minors. To shed more light on the problem of underage alcohol sales, unobtrusive research approaches such as mystery shopping are indispensable and are being used by police departments and other institutions in several countries (e.g., UK, Sweden) to enforce licensing legislation and compliance. Community mobilization and training of staff at supermarkets have shown promising results to decrease alcohol sales to underage youngsters but active and consistent enforcement of policies and engagement of sellers through educational programs is needed to effectively prevent adolescents’ alcohol use. The incorporation of these research approaches should be considered by municipalities in Spain for community mobilization and prevention of alcohol sales to minors.

## Figures and Tables

**Table 1:** Frequency of vendors’ compliance with age limitations for alcohol sales in supermarkets with younger minors (14–15 years old). Compliance was assessed through three indicators: whether cashiers ask for age and/or ID card, and alcohol sales to minors.

Type of Intervention	Control Group		Training of Trainers		Training of Trainers and Vendors Trainings	
	2018 (*n* = 4)	2020 (*n* = 5)	2018 (*n* = 14)	2020 (*n* = 4)	2018 (*n* = 10)	2020 (*n* = 6)
Asking age	50.0% (*n* = 2)	60.0% (*n* = 3)	14.3% (*n* = 2)	50.0% (*n* = 2)	10.0% (*n* = 1)	50.0% (*n* = 3)
Asking ID	100% (*n* = 4)	60.0% (*n* = 3)	28.6% (*n* = 4)	75.0% (*n* = 3)	30.0% (*n* = 3)	83.3% (*n* = 5)
Alcohol sales	25.0% (*n* = 1)	60.0% (*n* = 3)	64.3% (*n* = 9)	25.0% (*n* = 1)	50.0% (*n* = 5)	83.3% (*n* = 5)

Chi-square analysis (or Fisher test when the conditions of application were not fulfilled). All the chi-square (or Fisher) analysis were not statistically significant.

**Table 2:** Frequency of vendors’ compliance with age limitations for alcohol sales in supermarkets with older minors (16–17 years old). Compliance was assessed through three indicators: whether cashiers ask for age and/or ID card, and alcohol sales to minors.

Type of Intervention	Control Group		Training of Trainers		Training of Trainers and Vendors Trainings	
	2018 (*n* = 11)	2020 (*n* = 11)	2018 (*n* = 18)	2020 (*n* = 23)	2018 (*n* = 16)	2020 (*n* = 16)
Asking age	9.1% (*n* = 1)	36.4% (*n* = 4)	11.1% (*n* = 2)	30.4% (*n* = 7)	0% (*n* = 0)	43.8% * (*n* = 7)
Asking ID	9.1% (*n* = 1)	45.5% (*n* = 5)	5.6% (*n* = 1)	13.0% (*n* = 3)	6.3% (*n* = 1)	68.8% ** (*n* = 11)
Alcohol sales	81.8% (*n* = 9)	63.6% (*n* = 7)	88.9% (*n* = 16)	73.9% (*n* = 17)	93.8% (*n* = 15)	31.3% ** (*n* = 5)

Chi-square analysis (or Fisher test when the conditions of application were not fulfilled). Significance levels, * *p* < 0.05 and ** *p* < 0.01.

**Table 3:** Visibility of warning signs in supermarkets visited in 2018 and in 2020 for all the intervention groups.

Type of Intervention	Year	
	2018	2020
Control group supermarkets	93.3% (*n* = 14)	75.0% (*n* = 12)
Training of Trainers supermarkets	43.8% (*n* = 14)	66.7% (*n* = 18)
Training of Trainers and vendors trainings supermarkets	61.5% (*n* = 16)	100% (*n* = 22) **

Chi-square analysis (or Fisher test when the conditions of application were not fulfilled). Significance levels, ** *p* < 0.01.
